# Scientific misconduct responsibility attribution: An empirical study on byline position and team identity in Chinese medical papers

**DOI:** 10.1371/journal.pone.0308377

**Published:** 2024-08-05

**Authors:** Xiaoting Peng, Dehua Hu, Yi Guo, Hao Jiang, Xunsheng Wu, Qingyuan Hu

**Affiliations:** 1 The Third Xiangya Hospital of Central South University, ChangSha, Hunan, China; 2 Department of Biomedical Informatics, School of Life Sciences, Central South University, Changsha, Hunan, China; 3 Shenzhen Health Development Research and Data Management Center, Shenzhen, Guangdong, China; Delta State University, NIGERIA

## Abstract

**Objective:**

The primary objective of this inquiry was to explore the nexus between authorship attribution in medical literature and accountability for scientific impropriety while assessing the influence of authorial multiplicity on the severity of sanctions imposed.

**Methods:**

Probit regression models were employed to scrutinize the impact of authorship on assuming accountability for scientific misconduct, and unordered multinomial logistic regression models were used to examine the influence of authorship and the number of bylines on the severity of punitive measures.

**Results:**

First authors and corresponding authors were significantly more likely to be liable for scientific misconduct than other authors and were more likely to be penalized particularly severely. Furthermore, a negative correlation was observed between the number of authors’ affiliations and the severity of punitive measures.

**Conclusion:**

Authorship exerts a pronounced influence on the attribution of accountability in scientific research misconduct, particularly evident in the heightened risk of severe penalties confronting first and corresponding authors owing to their principal roles. Hence, scientific research institutions and journals must delineate authorship specifications meticulously, ascertain authors’ contributions judiciously, bolster initiatives aimed at fostering scientific research integrity, and uphold an environment conducive for robust scientific inquiry.

## Introduction

Research integrity serves as the foundational pillar of innovation in science and technology. The proliferation of scientific misconduct has eroded confidence significantly within the research community, in the medical domain, and among the general public alike. Scientific misconduct not only imperils the robust progression of scientific inquiry but also influences public perception of scientific and technological professionals, thereby impeding the establishment of a robust framework for scientific research integrity [1].

Among the many fields of academic research, the risk of scientific misconduct in the medical field is very high [2], especially when it comes to authorship and the attribution of responsibility for scientific misconduct. In 2021, China’s National Health Commission, Ministry of Science and Technology, and State Administration of Traditional Chinese Medicine collectively promulgated the Code of Integrity and Related Conduct for Medical Research. This regulatory framework underscores the requirement that authorship attributions across papers, publications, patents, and other scholarly endeavors adhere to a hierarchical ranking reflective of their respective contributions to research outcomes [3]. Authorship attribution not only pertains to the allocation of academic recognition and the identification of accountable contributors to scholarly works but also plays a pivotal role in ensuring fairness and precision in evaluating individuals, institutions, and disciplines [4]. Hence, elucidating the nexus between authorship and accountability for scientific misconduct emerges as a matter of paramount importance in academic discourse.

The association between authorship and liability for scientific misconduct remains inadequately examined in current scholarly investigations. Therefore, this study employs quantitative analysis to elucidate the association between authorship and liability for scientific misconduct, to furnish a foundational framework for bolstering research integrity, refining talent evaluation mechanisms, and safeguarding the integrity of the scientific research ecosystem.

## Literature review

### Scientific misconduct

The pervasiveness of scientific misconduct in the medical domain not only jeopardizes patient welfare but also causes the erosion of professional ethics among healthcare practitioners [5]. The 213 typical cases of scientific misconduct identified from 1997 to 2017 encompassed various forms, including plagiarism, data falsification, multiple submissions, information falsification, thesis purchase, and embezzlement of funds [1]. Medical literature faces multifaceted challenges, including instances of multiple submissions, unmonitored use of medical research grants, lapses in adherence to medical ethics codes, data integrity concerns, and notable risks to scientific integrity posed by improper attribution [2]. The lack of transparency in the investigation and decision-making procedures regarding scientific misconduct exacerbates the arbitrary nature of penalties imposed for such transgressions [6].

### Authorship

Scientific collaboration is advancing extensively, with high levels of engagement across multiple scales and hierarchical tiers. Collaborative research publications serve as a key avenue for disseminating scientific findings, although encountering inherent challenges such as those pertaining to authorship attribution [7]. The International Committee of Medical Journal Editors has delineated four simultaneous criteria required for authorship attribution in scientific publications [8]. In academic medical literature, scholarly papers typically feature two or more authors, yet frequently, authorship attribution does not reflect individual contributions to the research endeavor accurately. Consequently, the first or corresponding author may bear responsibility for the paper’s research content [9], thereby increasing the likelihood of their accountability for scientific misconduct. First and corresponding authors are closely associated with research performance evaluations and accolades, serving as pivotal assessment criteria in the career advancement trajectory [10]. Moreover, research has investigated the characteristics of authorship order from both a gender-specific [11] and national standpoint [12].

### Author position and scientific misconduct link

The positioning of an author’s name implies the significance of their contribution to the scientific paper, with the severity of penalties for scientific misconduct often contingent on the positioning of the author’s name as well.The correlation between authorship placement and instances of scientific misconduct is predominantly examined through analyses of relational patterns, impact severity, and resolution strategies.

In examining relational patterns, a U-shaped relationship delineates the degree of contribution relative to the positioning of the authors’ names. Typically, first authors and last authors contribute more substantially to tasks compared to middle authors [13].

Research has demonstrated that hierarchical authorship order complicates the delineation of an individual’s contribution to a scientific manuscript, thereby heightening the challenge of pinpointing the author whose contribution is deemed most significant [14,15]. The absence of consensus regarding “fair authorship” practices, coupled with the prevalence of phenomena such as “ghost authorship” and “gift authorship” [16], poses challenges in reflecting the genuine contributions of authors accurately in authorship attribution [17]. Furthermore, the escalating frequency of collaborative authorship teams exacerbates the complexity of discerning individual contributions [18], albeit with minimal impact [19]. First authors, corresponding authors, and senior authors are disproportionately associated with accountability for scientific misconduct compared to middle authors [20].

In addressing these challenges, extant research offers various solutions from the vantage points of programs, authors, and journals. From a project perspective, it is imperative to delineate task responsibilities associated with various authorship positions in the project and identify the requisite actions in diverse scenarios [21]. Authors were categorized, from their perspective, into primary authors, senior or supervisory authors, and contributing authors [22]. From a journal’s perspective, journal editorial boards should focus on developing and hiring professionals who are well versed in research standards to prevent research misconduct [23]. Additionally, scholars argue that prioritizing education as the cornerstone and using punishment as a supplementary measure is a crucial safeguard in upholding academic integrity [24].

Authorship attribution constitutes a critical research focal point in fostering a robust environment conducive to upholding integrity in medical research endeavors. Existing research concentrates predominantly on aspects such as research misconduct, authorship attribution, and the interplay between the two. However, there remains a paucity of investigation into authorship attribution and its association with accountability for research misconduct. Hence, this study aims to quantify the accountability for scientific research misconduct and elucidate the nexus between authorship attribution and responsibility for scientific research misconduct. Such endeavors seek to furnish a theoretical framework for enhancing the integrity of medical research.

## Materials and methods

### Data materials

The Ministry of Science and Technology of the People’s Republic of China, henceforth referred to as the Ministry of Science and Technology (MOST) through its Department of Scientific Research Integrity Construction, assumes responsibility for overseeing and providing guidance to various entities, including scientific and technological management agencies at all levels, in the scientific misconduct of cases related to scientific research misconduct [25]. On August 23, 2021, the Joint Conference on Scientific Research Integrity Construction resolved to institute a mechanism for notifying scientific research misconduct cases, aimed at publicly disclosing the outcomes of investigations and associated penalties [26].

The data collection period for this study concluded on May 30, 2023, during which the MOST issued a total of 22 sets of outcomes regarding medical research misconduct cases. The available results encompass 22 iterations of medical research misconduct cases, comprising a total of 553 English-language medical papers, spanning the period from June 8, 2021, to May 23, 2022.

### Methods

Initially, the data underwent a deduplication process. This process involved cross-referencing the titles of medical papers, resulting in the removal of three duplicate records. Subsequently, a sample comprising 550 medical papers, identified by the MOST as involving scientific misconduct, was obtained for this study. In total, these papers involved 2584 authors.

In the second stage, authorship attribution and the severity of penalties were quantified. Author names serve as indicators of authorship in medical papers; hence, this study identified authorship based on the authorship order. Authorship was classified into four categories: first author, second author, corresponding author, and other authors (referring collectively to authors other than the first, second, and corresponding authors). Numbers 4 to 1 represent: first author, second author, corresponding author, and other authors. In cases where an author served as both the first and corresponding author, the authorship was designated as the first author. If multiple authors were designated as cofirst authors, all cofirst authors were acknowledged as first authors. Similarly, if multiple authors were designated as co-corresponding authors, all co-corresponding authors were acknowledged as corresponding authors.

Following categorization, the degree of punishment was classified into five categories: not punished, less severely punished, somewhat severely punished, severely punished, and especially severely punished. Numbers 1–5 represent: not punished, less severely punished, somewhat severely punished, severely punished, and especially severely punished. Combined with the authorship and the types of penalties, the degree of punishment is categorized into a total of five types: not punished, less severely punished, somewhat severely punished, severely punished, and especially severely punished. The types of penalties corresponding to the degree of punishment are shown below.

A penalty level of “not punished” means that no penal measures have been taken. If the degree of punishment is “less severely punished” this means that it is subject to “research integrity caveat” and “stop scientific and technological activities supported by financial funds such as science and technology programs (special projects, funds, etc.), projects, with a deadline for rectification.” A penalty level of “somewhat severely punished” means that the person has been subjected to a penalty. In cases where a period of cancellation or prohibition is involved, the period is 3 years or less. A “severely punished” means that the penalty is the same as the “somewhat severely punished” category but involves a period of annulment or interdiction for 3 to 5 years. An “especially severely punished” means that the penalty is the same as the “somewhat severely punished” but involves a period of annulment or prohibition for 5 years or more.

Finally, a descriptive comparison was conducted to examine the relationship between authorship and responsibility for scientific misconduct, both with and without responsibility being attributed. Additionally, various regression methods were used to evaluate the probability of scientific misconduct in different authorship, and the influence of the number of authors on the occurrence of scientific misconduct was explored. The data analysis was performed using Stata 17.0 software.

This study addresses three primary issues:

First, it examined the impact of authorship on assuming responsibility for scientific misconduct. Here, the variable representing the assumption of responsibility for scientific misconduct served as the dependent variable, constituting a binary variable with values of 0 and 1 authorship was designated as the independent variable, encompassing categories such as first author, second author, corresponding author, and other author. The reference category for authorship was defined as other authors. Probit regression analysis was conducted to analyze the relationship.

Second, this study examined the influence of authorship on the severity of punishment. An unordered multicategorical logistic regression analysis was performed, with the degree of punishment serving as the dependent variable. “More punished” was designated as the reference category, while authorship was considered the independent variable.

Third, this study investigated the impact of the number of authors’ names on the degree of responsibility and the severity of penalties for scientific misconduct.

## Results

### Basic information

#### Categories of scientific misconduct

Table 1 presents the categorical statistics of scientific misconduct observed in 550 medical papers. Each medical paper may exhibit one or multiple types of scientific research misconduct. The classification of scientific research misconduct adheres to the guidelines outlined in the Rules for Investigation and Handling of Scientific Research Misconduct issued by the MOST in 2022. Among the 550 medical papers examined, seven major categories of scientific research misconduct were identified. The most prevalent forms of scientific misconduct, occurring 300 times, involved the fabrication of the research process, falsification of research results, sale and purchase of experimental research data, and forgery and tampering with experimental research data, charts, conclusions, test reports, or user use reports.

**Table 1:** Classification of scientific misconduct in 550 medical papers.

Num	Scientific misconduct	Times
1	Plagiarism, which denotes the unauthorized reproduction or misappropriation of other individuals’ research findings or project applications.	10
2	Fabrication of the research process, falsification of research outcomes, trading of experimental research data, and the falsification and alteration of experimental research data, diagrams, conclusions, test reports, or user reports.	300
3	Activities such as buying, selling, composing, or submitting papers or project acceptance materials on behalf of an applicant, and the creation of fictitious peer review experts and their evaluations.	251
4	To obtain the approval of scientific research activities, scientific and technological plans (special projects, funds, etc.), scientific research funds, awards, honors, job titles, etc. by intentionally providing false information and other fraudulent means, or by resorting to improper means such as solicitation, bribery, and interest exchange.	0
5	Acquisition of approval for scientific and technological ethical review through means of falsification, forgery, or tampering with documents intended for such review.	3
6	Authors’ names on documents lacking substantial academic contributions, in addition to other infringements on thesis, award, patent, and other authorship norms.	183
7	Instances of repeated publication, citation of literature unrelated to the paper’s content, solicitation of specific literature citations from authors unnecessarily, and various other infractions against academic publishing standards.	125

#### Types of punitive measures

Table 2 shows the statistics regarding the types of sanctions imposed on 2584 authors of medical papers. Each author may incur one or multiple types of sanctions. The penalty involving “disqualification as a nominator or recommender, nominee or recommender, assessment expert, etc. for a certain period” was the most prevalent, occurring 855 times.

**Table 2:** Punitive measures for 2584 medical authors.

Num	Types of penalties	Times
1	Integrity Admonishment Talk in Scientific Research.	545
2	Public notification within a certain range.	527
3	Stop scientific and technological activities supported by financial funds such as science and technology programs (special projects, funds, etc.), and projects, with a deadline for rectification.	78
4	Termination or withdrawal of scientific and technological activities supported by financial funds such as scientific and technological programs (special projects, funds, etc.), projects obtained by using scientific and technological research misconduct, recovery of the balance of funds, and recovery of allocated financial funds.	304
5	Prohibited for a certain period from undertaking or participating in scientific and technological activities supported by financial funds such as science and technology programs (special projects, funds, etc.).	252
6	Revocation of academic awards, honors, etc., recovery of prizes obtained through scientific research misconduct, and revocation of job titles obtained through scientific research misconduct.	23
7	A certain period to cancel the application or declaration of scientific and technological awards, scientific and technological talent titles and job titles, promotion, and other qualifications.	499
8	Cancellation of the titles of high-level experts, such as academicians, which have been obtained, and the qualifications of members of academic organizations, such as societies, associations, research societies, and academic and degree committees, that are performing academic work.	260
9	Disqualification as a nominator or recommender, nominee or recommender, assessment expert, etc. for a certain period.	855
10	Reduction of enrollment or suspension of enrollment of graduate students for a certain period up to disqualification of graduate student instructors.	359
11	Recorded in the database of serious violations of scientific research integrity.	11

### Number of authors’ names on medical papers

**Fig 1:**
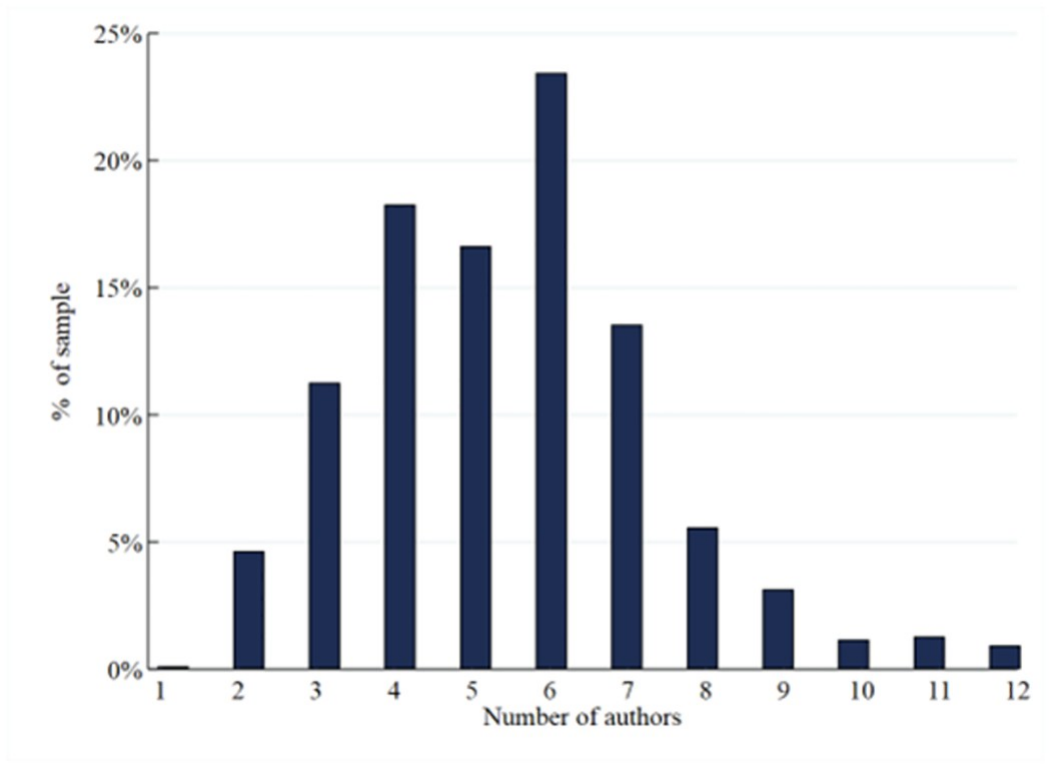
Distribution of the number of author attributions. The horizontal coordinate represents the number of authors in the 550 articles involving scientific misconduct, and the vertical coordinate represents the percentage of authors in the 550 articles involving scientific misconduct.

Fig 1 illustrates the distribution of the number of author names per medical paper, presenting a mean of 5.45 and a standard deviation of 1.88. Notably, a mere three articles (0.1%) feature a sole author. There is a wide range of author names, with the highest count of 12 author names observed across 550 medical papers. Specifically, the distribution reveals a concentration of author names at 6 (23.5%), followed by 4 (18.3%), and then 5 (16.6%).

### Authorship and liability in scientific misconduct

**Fig 2:**
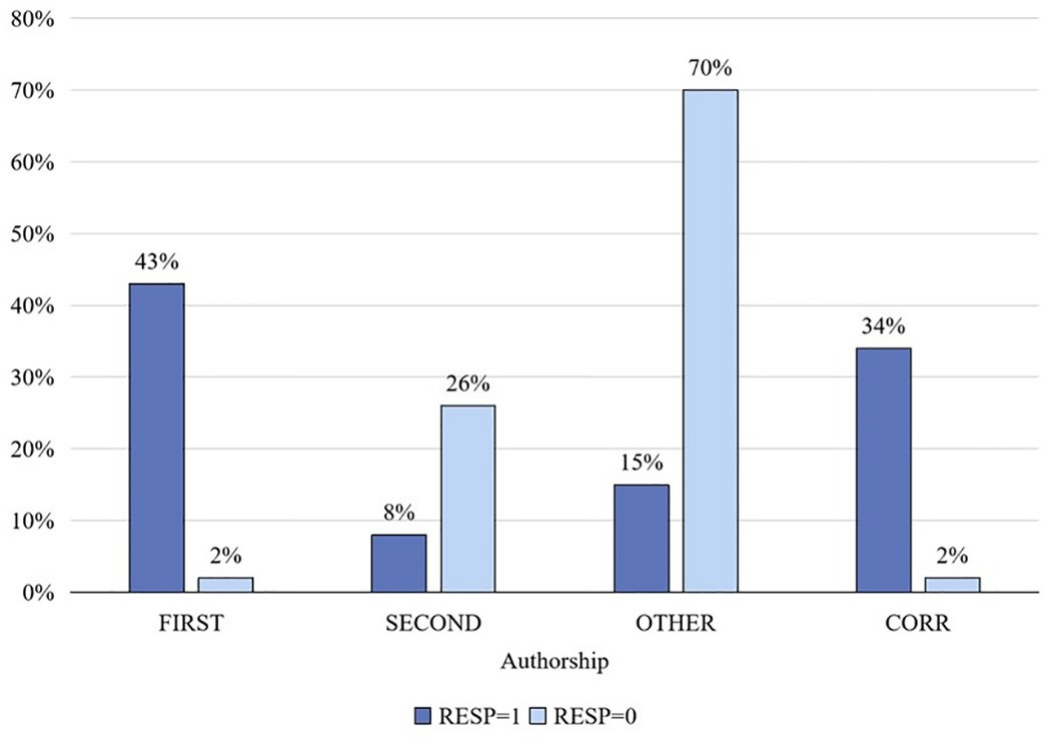
Distribution of authorship on responsibility for scientific misconduct. Abbreviations: FIRST = first author; SECOND = second author; OTHER = other author; CORR = corresponding author. The abscissa represents authorship, and the ordinate represents percentage. RESP = 1: Represents the percentage of each authorship category among 1,462 authors responsible for scientific misconduct, shown in dark blue; Resp = 0: 1,122 authors not responsible for scientific misconduct, percentage for each authorship category, shown in light blue.

Fig 2 shows the distribution of authorship attributions regarding the assumption of responsibility for instances of scientific misconduct. Notably, within the subset of authorships where responsibility for scientific misconduct was disclaimed, the predominant contributors were the second author and other authors, representing 26% and 70% of the total, respectively. Conversely, in cases where accountability for scientific misconduct was acknowledged, primary responsibility was predominantly shouldered by the first authors and corresponding authors, comprising 43% and 34% of the total, respectively.

The association between authorship and the acknowledgment of responsibility for scientific misconduct, treated as a binary variable (0 denoting absence of responsibility and 1 indicating accountability), was examined using a chi-square test. The analysis revealed a statistically significant relationship between authorship and the acceptance of responsibility for scientific misconduct (X^2^ = 1387.2, R = 0.732, *P* < 0.001),it indicates a significant relationship between these variables.

### Authorship credit and liability in scientific misconduct

Probit regression analysis was conducted to assess the average marginal effect, with authorship serving as the independent variable and authors’ acknowledgment of responsibility for scientific misconduct as the dependent variable. The reference category for authorship was designated as “other authors” within authorship. Table 3 presents the impact of authorship and the number of authors on the propensity for assuming responsibility in instances of scientific misconduct.

**Table 3:** Authorship attributions’ liability: Probit regression model.

Model	Authorship	Mfx. at mean	Coef.	SE	Z value	P value	95% *CI*
1	FIRST	0.5472	2.694	0.016	34.570	0.000	2.48–2.91
	CORR	0.5041	2.482	0.015	33.410	0.000	2.27–2.69
	SECOND	0.0412	0.203	0.016	2.570	0.010	0.048–0.36
2	FIRST	0.5381	2.635	0.017	31.330	0.000	2.41–2.86
	CORR	0.4838	2.433	0.016	30.650	0.000	2.22–2.65
	SECOND	0.0300	0.148	0.017	1.780	0.076	-0.02–0.31
	Number of authors	-0.0007	0.038	0.004	-2.090	0.036	-0.07–0.00

Abbreviations: FIRST = first author; CORR = corresponding author; SECOND = second author.

The probit regression analysis examining the relationship between authorship and the assumption of responsibility for scientific misconduct yielded statistically significant results (X^2^ = 0.4641, *P* < 0.001). Notably, first authors showed a significantly higher likelihood of being attributed culpability for scientific misconduct compared to other authors, with a mean marginal effect of 54.72% (95% *CI*: 2.48–2.91, *P* < 0.001). Similarly, the probability of corresponding authors being held accountable for scientific misconduct over other authors was observed at 50.41% (95% *CI*: 2.27–2.69, *P* < 0.001). Conversely, the likelihood of second authors assuming responsibility for scientific misconduct was markedly lower, at 4.12% (95% *CI*: 0.48–0.36, *P* < 0.05). See model 1.

The regression model exploring the influence of authorship and the number of authors’ bylines on the assumption of responsibility for scientific misconduct remained statistically significant after the inclusion of the control variable representing the number of authors’ bylines (X^2^ = 0.4563, *P* < 0.001). The acceptance of responsibility for scientific misconduct was found to be impacted by the number of authors’ bylines (95% *CI*: −0.07–−0.002, *P* < 0.05), indicating that with each additional author, the likelihood of assuming responsibility for scientific misconduct decreased by 0.07%. See model 2.

Furthermore, authorship exerted a discernible effect on the likelihood of assuming responsibility for scientific misconduct. In comparison to other authors, the probability of first authors bearing responsibility for scientific misconduct was notably higher, at 53.81% (95% *CI*: 2.41–2.86, *P* < 0.001). Similarly, corresponding authors exhibited a substantial likelihood of assuming responsibility, with a probability of 48.38% (95% *CI*: 2.22–2.65, *P* < 0.001). Conversely, the involvement of second authors did not yield statistically significant effects on the assumption of responsibility for scientific misconduct (95% *CI*: −0.02–0.31, *P* > 0.05).See model 2.

### Authorship influence on punishment level

An unordered multicategorical logistic regression analysis was performed, employing the degree of punishment as the dependent variable and designating “SSP” as the reference category. Authorship, with “OTHER” as the reference group, was examined as the independent variable. The analysis revealed a statistically significant effect of authorship on the degree of punishment (X^2^ = 0.296, *P* < 0.001), indicating a significant overall model fit (Table 4).

**Table 4:** Authorship-punishment link: Unordered multicategorical logistic model.

Degree of Punishment	Authorship	*β*	SE	Z value	OR value	P value	95% *CI*
NP	FIRST	−4.422	0.360	−12.290	0.012	<0.001	-5.127 − -3.717
	SECOND	−0.422	0.368	−1.140	0.656	0.252	-1.143 − 0.300
	CORR	−4.359	0.336	−12.960	0.013	<0.001	-5.019 − -3.700
LSP	FIRST	−1.691	0.309	−5.470	0.184	<0.001	-2.297 − -1.085
	SECOND	−0.302	0.389	−0.780	0.740	0.438	-1.063 − 0.460
	CORR	−1.306	0.290	−4.510	0.271	<0.001	-1.874 − -0.738
SP	FIRST	2.159	0.348	6.210	8.663	<0.001	1.478 − 2.840
	SECOND	0.513	0.473	1.080	1.670	0.278	-0.415 − 1.441
	CORR	1.529	0.344	4.450	4.614	<0.001	0.855 − 2.203
ESP	FIRST	2.540	0.471	5.400	12.675	<0.001	1.617 − 3.462
	SECOND	1.099	0.598	1.840	3.000	0.066	-0.073 − 2.270
	CORR	1.946	0.469	4.150	7.000	<0.001	1.026 − 2.866

Abbreviations: FIRST = first author; CORR = corresponding author; SECOND = second author.

The coefficients associated with first and corresponding authors show a significant negative association with the category “NP” compared with “SSP” This signifies that in cases of scientific misconduct culpability, first and corresponding authors face an elevated likelihood of receiving a “SSP”. To elaborate, the probability of the first author receiving no penalty is substantially lower, by 98.8%, than facing a “SSP,” Similarly, corresponding authors are 98.7% less likely to avoid penalties altogether and are more prone to receiving a “SSP” instead.

The coefficients associated with first and corresponding authors exhibit a statistically significant negative association with the likelihood of receiving “LSP” penalties compared to “SSP” penalties. This suggests that when confronted with liability for scientific misconduct, both first and corresponding authors face an elevated risk of being subjected to “SSP” penalties. First authors showed an 81.6% reduced likelihood of receiving the designation “LSP” compared to “SSP,” while corresponding authors showed a 72.9% diminished probability of being categorized as “LSP” rather than “SSP.”

The coefficients for the first author and corresponding author are significantly positive compared to the coefficients for “SP” and “SSP.” This implies that when confronted with allegations of scientific research misconduct, both the first author and corresponding author face an elevated risk of being subjected to “SP.” Specifically, the probability of the first author receiving “SP” is 766.3% higher than “SSP.” Similarly, the likelihood of the corresponding author receiving “SP” is 361.4% higher than “SSP.”

The coefficients associated with first and corresponding authors demonstrate a statistically significant positive correlation with the category of “ESP” in comparison to “SSP.” This indicates that both first and corresponding authors face an increased likelihood of being subjected to “ESP” when confronted with allegations of scientific misconduct. First authors showed a 1167.5% heightened probability of receiving the designation “ESP” compared to “SSP” while corresponding authors displayed a 600.0% increased likelihood of being categorized as “ESP” rather than “SSP.”

### Author count impact on penalization degree

We used the degree of punishment as the dependent variable, selecting “SSP” as the reference group, and using the number of authors’ bylines as the independent variable for the unordered multicategorical logistic regression analysis (Table 5). The effect of the number of authors’ bylines on the degree of punishment is statistically significant (X^2^ = 0.043, *P* < 0.001), and the model is significant as a whole. The positive coefficients for “NP” and “LSP” and the negative coefficient for “ESP” indicate that the greater the number of authorship bylines, the more likely it is that the author team will not be penalized (i.e., “NP”) or “LSP,” and less likely to be “ESP.” Compared to a “SSP,” the author team is 42.5% more likely to be “NP” or “LSP,” 33.4% more likely to be “ESP,” and less likely to receive a “LSP.” The probability of receiving an “ESP” is 14.8% lower. In other words, the number of authors affects the degree of punishment of the author team.

**Table 5:** Authorship-liability regression: Disordered multicategorical model.

Degree of Punishment	β	SE	Z value	OR value	P value	95% *CI*
NP	0.354	0.057	6.230	1.425	<0.001	0.243 − 0.465
LSP	0.288	0.060	4.780	1.334	<0.00	0.170 − 0.406
SP	-0.024	0.059	-0.420	0.976	0.678	-0.139 − 0.091
ESP	-0.160	0.065	-2.460	0.852	0.014	-0.288 − -0.033

Abbreviations: NP = not punished; LSP = less severely punished; SP = severely punished; ESP = especially severely punished.

## Discussion

In this investigation, adopting an authorship-centric approach, we leveraged datasets on instances of medical research integrity breaches disseminated by the MOST. While retraction statements on academic literature websites primarily emphasize detailing the specific scientific misconduct violations in the paper, the MOST’s dataset on medical research misconduct case outcomes provides additional clarification regarding the attribution of authorship, authorship roles, and individual accountability in the author team implicated in the scientific misconduct. Hence, the dataset on the outcomes of medical research misconduct cases provided by the MOST facilitates a comparative analysis of the variations in liability for scientific misconduct across different authorship roles. Previous research has explored the characteristics of medical academic misconduct papers using data derived from the MOST’s medical research integrity cases. These investigations have examined various aspects, including the characteristics of authors, papers, retraction time lag, instances of academic misconduct [27], and the distribution of journals and institutions associated with the publication of academic misconduct papers [28]. Consequently, this study endeavors to investigate the relationship between authorship and accountability for scientific misconduct, employing data sourced from the outcomes of the MOST’s medical research misconduct cases.

By applying probit regression and unordered multiclassified Logit regression techniques, we undertook an analysis to discern the ramifications of authorship on the attribution of culpability for scientific malfeasance and the severity of ensuing disciplinary measures. The discoveries gleaned from this inquiry are described below. The distribution of authors’ names clusters predominantly around four, five, or six individuals. Notably, the variability in the number of author names observed in this study closely mirrors the findings reported by Hussinger and Pellens [20] concerning a sample of medical papers, thereby underscoring the external validity of the sample employed in this study.

As scientific endeavors advance, the need for a broader spectrum of expertise and augmented resources becomes urgent to navigate the nuances of interdisciplinary research [29]. Empirical investigations have underscored the pervasive global trend of scholarly works being authored by expansive consortia, a phenomenon mirrored in the scientific landscape of China wherein research output is predominantly steered by collaborative endeavors comprising more than four researchers. This proliferation of large-scale collaborative ventures not only reflects the diversification of scientific inquiry but also aligns with the strategic dispositions of funding agencies. Notably, the National Natural Science Foundation of China tends to channel its financial allocations to bolster the undertakings of extensive research collectives, particularly in the domain of medical research. Moreover, scholarship emanating from such expansive collaborations garners a superior mean citation count [30]. Irrespective of national boundaries, scholars universally need to cultivate ethical precepts in research, a proficient comprehension of pertinent legislative frameworks, and an unwavering commitment to upholding the fundamental principles of research integrity.

Authorship exerts a robust correlation with the attribution of responsibility for scientific misconduct. The onus associated with scientific malfeasance is primarily on the first author and corresponding author, with the second author assuming a secondary responsibility in this regard. Studies have shown that a positive association between factors indicating the severity of misconduct or a pattern of misbehavior in respondents and the severity of ORI administrative actions [31]. Scholarly inquiry has delineated a nexus between the prevalence of scientific misconduct and various factors, including the imperative to publish, career advancement imperatives, and access to research funding in the biomedical domain, with a discernible higher incidence observed among researchers of Asian descent [32]. Notably, the attainment of a requisite number of publications as the first author often constitutes a graduation prerequisite for graduate students across numerous academic institutions. These compounding factors exacerbate the propensity for scientific misconduct, further compounded by the prevailing perception among scientists that the first author predominantly spearheads the research endeavor and plays a pivotal role in the manuscript composition process [33]. This acknowledgment underscores the marked esteem accorded to first authorship within academic circles. Beyond conferring heightened visibility and project funding opportunities upon authors, first authorship augments their standing within the academic community, particularly enhancing their prospects in academic evaluations and career progression [34]. Additionally, the corresponding authorship, as a distinctive designation, is often equated with the first author’s status in the assessment of scholarly productivity across various domestic research institutions [35]. Moreover, influenced by the prevailing social milieu characterized by a penchant for rapid achievements and gains, along with a culture of braggadocio, both the first author and corresponding author frequently harbor aspirations of swiftly bolstering their prestige within the academic fraternity and reaping commensurate benefits and returns from their endeavors [36]. Consequently, it can be inferred that these two forms of authorship harbor a potent impetus for the manifestation of scientific misconduct.

In instances of culpability for scientific misconduct, the roles of first author and corresponding author are particularly susceptible to severe punitive measures. In the domain of scientific inquiry, assuming the positions of first and corresponding authors entails not only significant responsibilities in averting research misconduct but also pivotal roles in safeguarding the authenticity and reliability of research outcomes. Traditionally, the first author assumes the lion’s share of substantive work, thus making a substantial contribution to and assuming primary accountability for the research findings. Meanwhile, corresponding authors primarily shoulder the responsibility of liaising with journal editors, peer reviewers, and other experts throughout the submission process, thereby making substantial contributions to the research outcomes [37]. Given the pivotal contributions of first and corresponding authors to the integrity of research findings, assuming the responsibility for ensuring the authenticity of experimental data and rectifying any deficiencies in medical manuscripts constitute primary obligations inherent to these authorial roles. Notably, individuals donning the mantle of first or corresponding author encompass a diverse spectrum of stakeholders, ranging from university presidents and vice presidents endowed with extensive research acumen to stalwarts within the academic community such as deans of secondary colleges and departmental chairs, alongside ordinary students and educators [36]. This cohort bears the collective responsibility of attesting to the veracity of research outcomes, as any lapses in this regard could precipitate a diminution in the overall credibility of the research enterprise.

While the number of authors exhibits a statistically significant influence on the severity of penalties levied against authorial, scholarship underscores that an escalation in author count augments the costs associated with investigating scientific misconduct. Collective punishment is becoming increasingly common, especially when detection methods used to prevent scientific misconduct are ineffective [38]. Moreover, the proliferation of authors amplifies the costs incurred in facilitating communication and coordination among authorial cohorts, thereby engendering challenges in managing the research process and ensuring quality control over research outcomes. In addition, the rapidly increasing number of author teams has become an obstacle to disseminating scientific research results. This dilemma has prompted certain esteemed journals to impose limitations on the number of authors per article, reflecting the conundrum of scientifically and judiciously evaluating individual contributions within expansive authorial ensembles. The deleterious ramifications of stigmatization, for scientific misconduct merely through association, can precipitate a decline in citation rates for articles authored by previously blameless collaborators, thereby impeding the pace of scientific advancement [38]. Hence, it behooves relevant research institutions to make concerted efforts to impart education and training initiatives geared toward enhancing researchers’ awareness of research integrity, thereby augmenting the overarching ethical ethos within the research community.

## Limitation

Our study underscore that both the first author and the corresponding author are held accountable for scientific misconduct, regardless of their specific roles. This indicates that primary responsibility for scientific misconduct lies with these authors. Our study has several notable limitations. Firstly, the sample is drawn from China, and further verification is needed to determine if it represents global research on scientific misconduct. Additionally, expanding the sample size and conducting follow-up studies after MOST publishes more cases can provide valuable insights. Secondly, our sample is limited to basic medicine and clinical research funded by MOST, thus not representing a wide range of fields such as physics, mathematics, engineering, and chemistry. Other researchers should be encouraged to investigate cases of scientific misconduct in these areas as well.
